# Yeast biofilm in food realms: occurrence and control

**DOI:** 10.1007/s11274-020-02911-5

**Published:** 2020-08-10

**Authors:** Giacomo Zara, Marilena Budroni, Ilaria Mannazzu, Francesco Fancello, Severino Zara

**Affiliations:** Department of Agricultural Sciences, University of Sassari, Sassari, Italy

**Keywords:** Adhesion, Biofilm, *Candida* spp., Extracellular matrix, Polyphenols, Quorum sensing, *Saccharomyces cerevisiae*

## Abstract

In natural environments, microorganisms form microbial aggregates called biofilms able to adhere to a multitude of different surfaces. Yeasts make no exception to this rule, being able to form biofilms in a plethora of environmental niches. In food realms, yeast biofilms may cause major problems due to their alterative activities. In addition, yeast biofilms are tenacious structures difficult to eradicate or treat with the current arsenal of antifungal agents. Thus, much effort is being made to develop novel approaches to prevent and disrupt yeast biofilms, for example through the use of natural antimicrobials or small molecules with both inhibiting and dispersing properties. The aim of this review is to provide a synopsis of the most recent literature on yeast biofilms regarding: (i) biofilm formation mechanisms; (ii) occurrence in food and in food-related environments; and (iii) inhibition and dispersal using natural compounds, in particular.

## Yeast biofilm

The ability of fungal species to adhere to and grow on different substrates or hosts is surprisingly broad: from human and plant tissues to food matrices, fuel lines, and even bare rocks (Fanning and Mitchell 2012; Rola et al. 2016). Most of the knowledge accumulated on fungal biofilms has been stimulated by the implications of fungi, such as *Cryptococcus*, *Aspergillus*, *Pneumocystis*, *Coccidioides* in human pathogenesis (Fanning and Mitchell 2012). Similarly, health issues related to the development of the yeast *Candida albicans* have stimulated the study of yeast biofilms (Lohse et al. 2018). An imprecise distinction has been made between yeasts and those dimorphic filamentous fungi that often produce abundant yeast-like growth. Notwithstanding this possible confusion, yeasts, whether ascomycetes or basidiomycetes, have been defined as single-cell microorganisms generally characterized by budding or fission as the primary means of asexual reproduction, and having sexual states that are not enclosed in fruiting bodies (Kurtzman et al. 2011). As with other microbial biofilms, that produced by yeast is a highly structured microbial community associated with or attached to a surface, upon which the microbial cells enclose themselves within a self-produced extracellular matrix (Wu et al. 2017). Yeast beneficial biofilms in the food realm have been also described, mostly in relation to *Saccharomyces cerevisiae*, such as those required for the ageing and maturation of special wines, fermented olives and dairy products.

### Genetic determinants of yeast biofilm formation

In the first step of biofilm formation, yeast cells adhere to each other and to both biotic and abiotic surfaces (Bojsen et al. 2012). Cell attachment is mediated by specific adhesion molecules, called adhesins, through amyloid-like or hydrophobic interactions (Lipke 2018). In *S. cerevisiae*, different proteins have been described that are involved in biofilm formation, including Flo11p (Zara et al. 2005), Hsp12p (Zara et al. 2002), Ccw14p (Moreno-García et al. 2018), Fig. 2p (Van Mulders et al. 2010), etc. Of these, Flo11p is directly involved in biofilm formation on solid or semisolid agar, on plastic surfaces, and on the air-liquid interface (Reynolds and Fink 2001; Zara et al. 2005). Differences in the biofilm-forming ability of *S. cerevisiae* strains have been related to the number of repeated sequences and the transcriptional levels of *FLO11* (Zara et al. 2009). *FLO11* transcription responds to nutritional and environmental stimuli through signals that activate different pathways, including the MAPK cascade, the cAMP-PKA pathway, and the TOR pathway, as well as the Cyc8p/Tup1p complex (Vinod et al. 2008; Nguyen et al. 2018). Whole-genome resequencing has enabled 155 loci to be identified that are highly divergent between 110 *S. cerevisiae* strains able to form biofilm vs. those unable to do so (Coi et al. 2017). These loci include the major regulators cAMP, *IRA1*, and the MAP-kinases *STE7*, *KDX1*, and *RGA2*. In *Candida*, the major regulatory mechanisms governing biofilm development involve both the MAPK and cAMP pathways, as well as transcriptional regulators such as Bcr1p and Tec1p (Gulati and Nobile 2016; Fox et al. 2015).

**Fig. 1:**
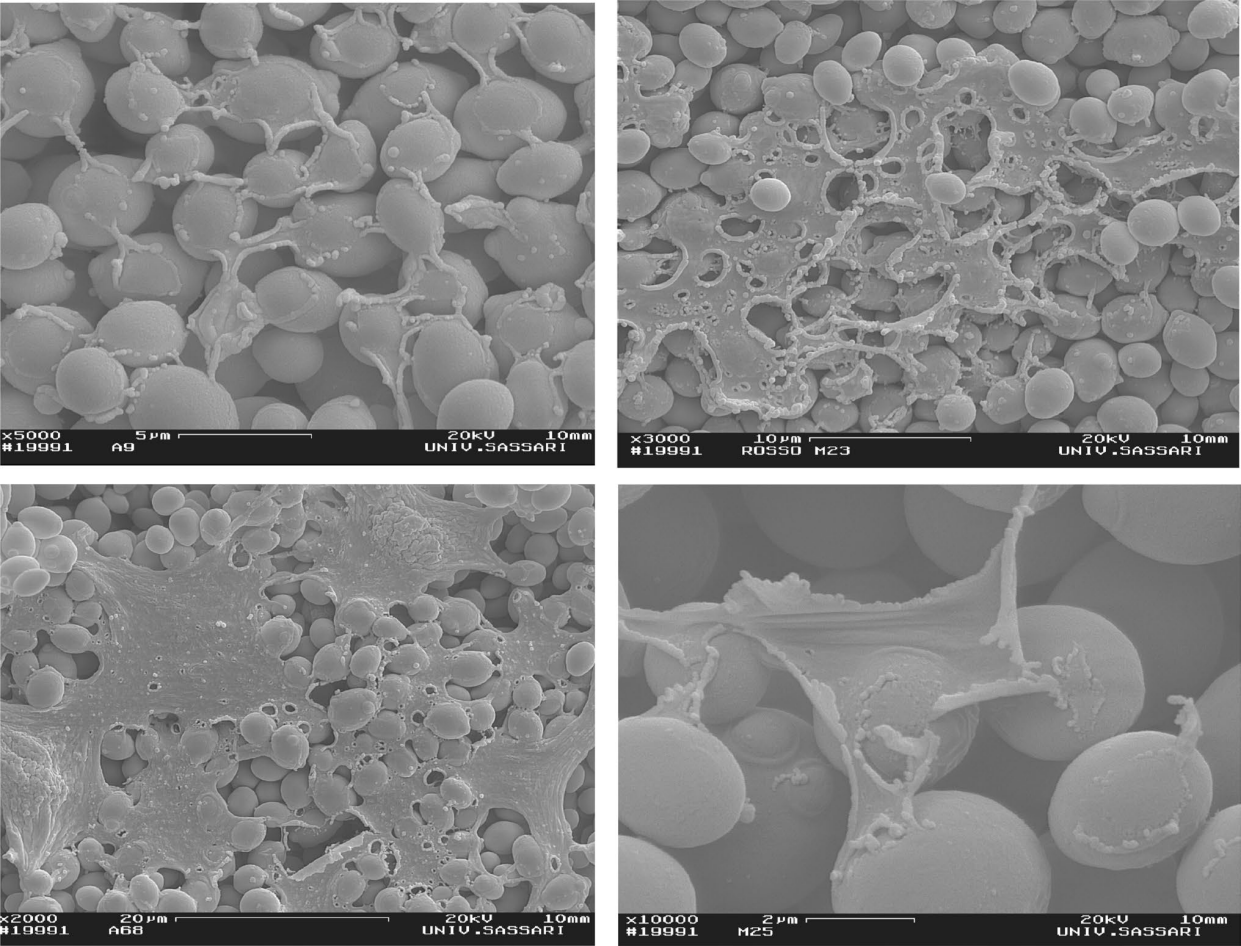
Extracellular matrix from different *S. cerevisiae* biofilm-forming yeast

Once yeasts cells have started to adhere to each other and to a surface, they proliferate, grow into filamentous forms (hyphae and pseudohyphae), and accumulate an extracellular matrix (ECM) (Vopálenská et al. 2010). Cells capable of forming a network of hyphae/pseudohyphae display stronger attachment to inert surfaces (e.g. stainless steel and plastic). In particular, the pseudohyphal content is directly correlated with biofilm strength and resistance, probably due to the higher amount of chitin in pseudohyphal cells (Paramonova et al. 2009). ECM is commonly made of carbohydrate, protein, lipid, and nucleic acid (Flemming and Wingender 2010; Faria-Oliveira et al. 2015). Karunanithi et al. (2010) suggested that in mature biofilms of *S. cerevisiae*, Flo11p forms part of the ECM, where it promotes a kind of cellular lubrication phenomenon that facilitates cellular motility. The ECM confers yeast cells a broad range of advantages, such as adhesion, cohesion, and mechanical properties, nutritional sources, enzymatic activities, and protection (Flemming and Wingender 2010) (Fig. 1).

### Nutritional factors that induce yeast biofilm formation

In most cases, the availability of nutrients, such as carbon and nitrogen sources, but also lipids, controls biofilm development. In *S. cerevisiae*, the ammonia permease Mep2p activates the MAPK and the cAMP–PKA pathways when low concentrations of diammonium phosphate are available (Vinod et al. 2008). Furthermore, TOR pathway activity switches off in response to increasing nitrogen concentrations (Vinod et al. 2008). Thus, biofilm formation and *FLO11* gene expression are enhanced in *S. cerevisiae* when nitrogen sources are scarce (Zara et al. 2011). Similarly, glucose starvation increases biofilm formation and *FLO11* expression through the cAMP–PKA and SNF1 pathways (Livas et al. 2011; Bojsen et al. 2012). Van Nguyen et al. (2020) reported that the inhibition of biofilm formation at high glucose concentration is mediated by increased Cyc8p-mediated *FLO11* repression. Zara et al. (2010) suggested that biofilm formation requires an energy input provided by reduced carbon sources. Indeed, greater biofilm formation was obtained by growing *S. cerevisiae* on glycerol, ethyl acetate, and ethanol. In addition to carbon and nitrogen compounds, lipids also play a major role in biofilm formation. In particular, the fatty acid residues of biofilm-forming strains of S. *cerevisiae* were shown to have a larger medium chain length and higher unsaturation levels than those in non-film‐forming strains (Zara et al. 2008). Zara et al. (2012) found that cerulenin, an inhibitor of the fatty acid synthase complex, prevents biofilm formation and *FLO11* transcription. Whole transcriptomic analysis revealed that a lack of lipid nutrients induces a stress response in biofilm-forming strains, leading to the induction of *PAU*s and *HSP*s gene families (Zara et al. 2019b). Similarly, biofilm cells of *C. albicans* differ from planktonic cells in their phospholipid, sterol, and sphingolipid content (Lattif et al. 2011). In accordance, sterol, fatty acid, and lipid metabolism pathways were found to be upregulated during biofilm formation (Lattif et al. 2008).

## Yeast biofilm in food realms

**Fig. 2:**
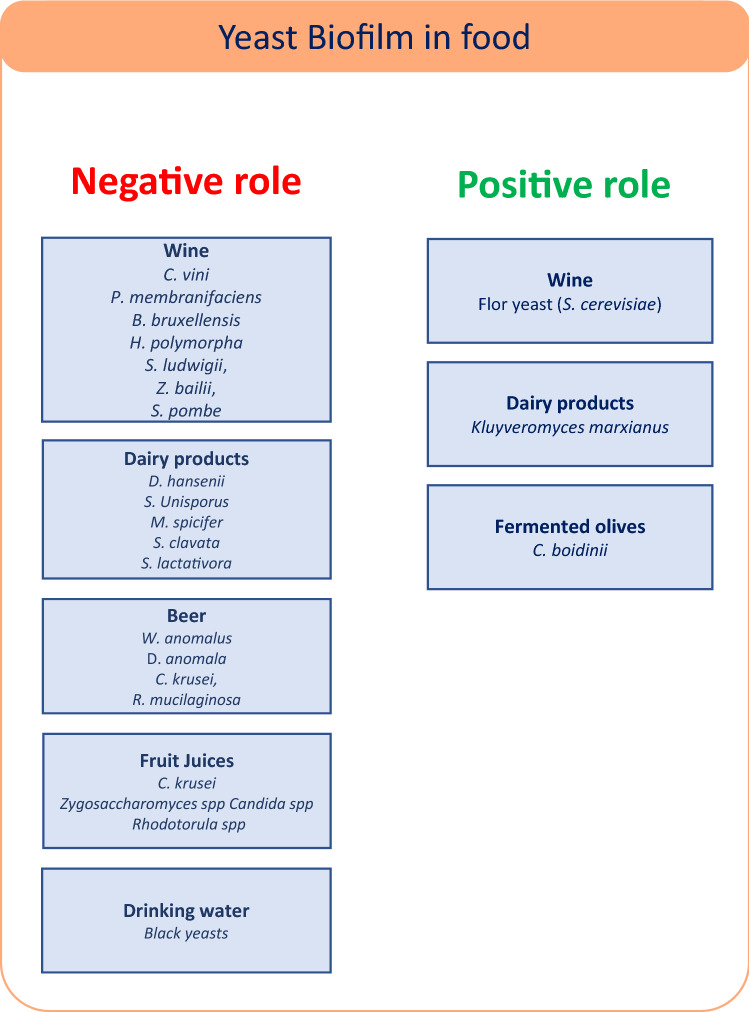
Negative and positive effects of yeast biofilm

Yeast are found in a range of fresh and processed foods: dairy, meat, fruit and vegetable products, syrups, honey, juices, soft drinks, alcoholic beverages, salad dressings, mayonnaise, confectioneries, jams, and bread (Deák 1991). The main source of microbial contamination in the production chain may be the processing plant itself, due to inadequate hygiene measures that can favor the formation of biofilms. Yeast contamination in food processing plants is likely due to aerosols and to splash and overspray during sanitation programs (Snyder and Worobo 2018). High levels of spoilage yeasts have also been found in floor drains (Snyder and Worobo 2018). An abundant growth of unwanted yeast, such as *Brettanomyces bruxellensis, Candida krusei, Candida parapsilosis, Debaryomyces hansenii, Kloeckera apiculata, Pichia membranaefaciens, Rhodotorula mucilaginosa, S. cerevisiae, Schizosaccharomyces pombe, Torulopsis holmii, and Zygosaccharomyces bailii*, can lead to problems in food quality and safety (Salo and Wirtanen 2005). In addition, many yeast species can develop as biofilm cells that are significantly more difficult to eradicate than planktonic cells (Brugnoni et al. 2007). Indeed, microbial biofilms cost the food industry several billion dollars every year due to product losses, reduced heat transfer, increased fuel consumption, and the excessive use of chemical agents for their control and removal (Chambers et al. 2006; Lyon et al. 2008; Srey et al. 2013). Below, some examples of how yeast biofilms spoil different foods and beverages, including drinking water, are provided (Fig. 2).

Yeasts have a central role in the fermentation of table olives and in the development of their sensorial features. In particular, yeasts contribute to olive debittering, through the activity of β-glucosidase, to the degradation of phytic complexes, and to the release of inorganic phosphorous, due to the production of enzymes such as phosphatases and phytases. Yeasts, particularly *Candida boidinii*, can co-aggregate on the surface of olives together with lactic acid bacteria (LAB) and establish poly-microbial biofilms (Arroyo-López et al. 2012; Benítez-Cabello et al. 2015). Recently, the presence of biofilm-forming yeast such as *Candida* spp. and *Pichia* spp. was also observed in olive oil by Santona et al. (2018), and although biofilm-forming yeast can make a positive contribution to olive fermentation and maturation (Camiolo et al. 2017; Porru et al. 2018), the consequences of their presence in olive oils remains open to debate (Santona et al. 2018; Fancello et al. 2020).

Another setting in which biofilms play an important role is that of dairy products. Microbial communities can influence the quality of dairy products in both a negative or positive way. Floor drains in dairy processing facilities are one of the niches where the formation of biofilms can occur. Due to their open system, floor drains are exposed to a wide range of microbes and nutrients and may also serve as a reservoir for food-borne pathogens. In particular, Schön et al. (2016) showed that floor drain communities were dominated by product-associated bacterial (e.g. *Lactobacillus kefiranofaciens*, *Streptococcus thermophilus*) and eukaryotic phylotypes (e.g. *Debaryomyces hansenii*, *Saccharomyces unisporus*). Vitzilaiou et al. (2019) found that the reverse osmosis membrane filtration elements from a whey water filtration unit were highly contaminated by biofilm-forming microbial populations, both before and after cleaning-in-place treatments. These microbial populations consisted of the budding yeasts *Sporopachydermia lactativora* in association with filamentous fungi (*Magnusiomyces spicifer* and *Saprochaete clavate*) and Gram-negative bacteria. In other instances, the development of a biofilm in dairy products contributes positively to the product. It has been showed that *Kluyveromyces marxianus* strains isolated from fermented goat’s milk were able to from a biofilm, and this yeast species also performs a useful role in the maturation of ‘Pecorino di Farindola’ and ‘Parmigiano Reggiano’ cheeses (Perpetuini et al. 2018, 2019).

With regard to alcoholic beverages, yeast biofilms are also present in the wine and beer industry. In the former, a well-known example of positive yeast biofilm is that represented by *S. cerevisiae* in Sherry *fino* (Spain) and Sherry-like wines, such as Szamorodni (Hungary), ‘Vin Jaune’ (France), ‘Vernaccia di Oristano’ (Italy), etc. At the end of the alcoholic fermentation, the so-called “flor strains” of *S. cerevisiae* rise to the surface of the wine and switch from fermentative to the oxidative metabolism (Legras et al. 2016). Specifically, yeast oxidize ethanol to produce acetaldehyde, which is the precursor of molecules responsible for the specific sensorial properties of biologically aged wines (Pozo-Bayón and Moreno-Arribas 2011). Contrary to these beneficial biofilms, the development of other oxidative yeast species on the surface of wine constitutes a common problem in alcoholic beverage industry. The visual manifestation is the formation of a film resulting from the repeated budding of mother and daughter cells that remain attached, forming chains that eventually develop into a thick biofilm over the entire surface of the wine (Fugelsang and Edwards 2007). This behavior has been ascribed to *Candida vini*, *P. membranifaciens*, and *Hansenula polymorpha*, which produce sensorially negative compounds. Other yeast species able to form a biofilm in wine belong to the fermentative species *Brettanomyces/Dekkera bruxellensis*, *Saccharomycodes ludwigii*, *Z. bailii*, and *S. pombe* (David-Vaizant and Alexandre 2018). Their development during wine storage leads to defects such as cloudiness or haziness, sediment production, off-odors, and off-tastes. In beer, yeast biofilms constitute a negative phenomenon. In breweries, yeast can form biofilms colonizing new surfaces or existing fungal or bacterial biofilms (Gattlen et al. 2011). In particular, yeasts have a high impact on alcohol-free beer and beer-mixed beverages, where they can be responsible for up to 90% of the spoilage incurred (Riedl et al. 2019). These beverages are characterized by chemical properties (a high sugar content and a low pH) that favors the attachment of first-stage yeast biofilm colonizers. *Wickerhamomyces anomalus* was reported to be the most important in the context of biofilm formation in breweries (Laitila et al. 2011). *Dekkera anomala* has also been reported to form biofilms in beer and wort, with strain-dependent biofilm production (Storgårds et al. 2006). *C. krusei, R. mucilaginosa*, *P. anomala*, *P. membranifaciens*, and *S. cerevisiae* are able to form biofilms on stainless steel surfaces and in the filling area, especially in the conveyor system near the filler (Storgårds et al. 2006). Similarly, *Brettanomyces* spp. are able to form a biofilm on plastic surfaces under conditions with low sugar concentrations (Joseph et al. 2007). Goode et al. (2010) investigated the efficacy of different cleaning procedures in reducing the formation of yeast biofilm in a brewery processing plant using NaOH based agents. However, biofilm formed of *D. anomala* was found to be resistant to many cleaning and disinfecting solutions, as well as antibiotics (Hutzler et al. 2012). Yeast biofilms also have a negatively effect upon fruit juice production lines. Yeast belonging to *Saccharomyces*, *Zygosaccharomyces*, *Candida*, and *Rhodotorula* spp. have all been isolated from biofilms on conveyor tracks, and on can and bottle warmers in the packaging department of fruit juice processing plants, due to their high resistance to thermal processing (Salo and Wirtanen 2005). Brugnoni et al. (2007, 2011) found that *C. krusei* isolated from a large-scale apple juice processing plant was capable of rapidly colonizing and covering stainless steel surfaces. Moreover, *C. krusei* was found in mixed biofilms subjected to varying hydrodynamic conditions commonly found in apple juice facilities (Brugnoni et al. 2014). Of particular significance, *C. krusei* biofilm was identified as potentially playing an important role in the survival and dissemination of *Escherichia coli* O157:H7 and *Salmonella enterica* in food-processing environments (Tarifa et al. 2017).

Finally, the negative role of yeast biofilm in drinking water systems needs to be considered. Black yeasts are commonly associated with water handling systems and form recalcitrant biofilms in filters, grates, and nozzle heads (De Hoog et al. 2006). In particular, mixed species microbial biofilms have been found in Drinking Water Distribution Systems (DWDS). DWDS are complex pipe networks that function as dynamic ecosystems where consortia of fungal-bacterial biofilms, attached to the inner pipe surfaces, are involved in a range of processes that ultimately determine the quality and safety of the delivered water. In particular, biofilm cells are responsible for the biocorrosion of metal pipes and their mobilization may affect the safety of bulk water (Husband et al. 2016).

## Control of yeast biofilm

Yeast biofilms are tenacious structures difficult to eradicate and to treat with the current arsenal of antifungal agents available. As Ramage et al. (2014) pointed out, fungal biofilm resistance involves both physical barriers and regulatory processes. Many genetic determinants of antifungal resistance are involved in the formation of ECM, the upregulation of drug efflux pumps, the activation of stress responses, and the modification of general cell physiology, such as a reduced growth rate (Fanning and Mitchell 2012; Gulati and Nobile 2016). Thus, the antifungal resistance of biofilms is multifactorial and heterogeneous, often related to specific developmental phases of biofilm formation. Furthermore, ECM composition and surface density greatly influence biocide effectiveness (Alvarez-Ordóñez et al. 2019). Biocides are generally employed by the food industries at concentrations the exceed the minimal inhibitory concentrations and should therefore be able to guarantee microbial inactivation. However, biocides, and other antimicrobials are less effective in inactivating microbes within biofilms than in the planktonic state (Cappitelli et al. 2014). It was reported that biofilm cells are up to 1000-fold more resistant to antifungal agents (Ramage et al. 2014). Considering the role of biofilms as a reservoir of potential spoilage and pathogenic microorganisms, much research effort is being devoted to improving the methods and strategies available to eliminate them from industrial settings or to developing novel inhibitory or removal tools that are more effective, economic, and sustainable.

### Natural compounds against yeast biofilms

Considering that the currently available antifungal agents usually have very little effect on yeast biofilms, novel active compounds derived from plants, lichens, algae, and microbes (fungi or bacteria) have been sought and described (Table 1).

**Table 1:** Compounds of natural origin with inhibiting activity against yeast biofilm

Origin/compound	Yeast species	Authors
Red pitaya (*Hylocereus polyrhizus*) polyphenolic fractions	Yeasts and molds	Tenore et al. (2012)
Cocoa, coffee, and beer polyphenols	*C. albicans*	Shahzad et al. (2014)
Olive leave extracts - oleuropein	*C. albicans*	Takó et al. (2020), Edziri et al. (2019)
Orange nano emulsion	*S. cerevisiae*	Sugumar et al. (2016)
Aldehydic terpenes	*Cryptococcus neoformans*	Kumari et al. (2019)
Clove and thyme - essential oils	*Candida* spp.	Rajkowska et al. (2019).
Laurel wood extract - procyanidins	*C. albicans*	Alejo-Armijo et al. (2017)
Mangrove - hydrolysable tannin	*C. albicans*	Glasenapp et al. (2019)
l-histidine	*S. cerevisiae*	Bou Zeidan et al. (2014)
Arginine, lysine, cysteine, tryptophan, phenylalanine, threonine	*S. cerevisiae*	Zara et al. (2019a)
N-acetyl cysteine	*C. albicans*	Abd et al. (2014)
Farnesol	*C. tropicalis*	Agustín et al. (2019)
Tyrosol	*Candida* spp.	Sebaa et al. (2019)
Killer toxins	*Candida* spp.	Tan and Tay (2011)

Plants comprise the largest source of compounds active against fungal biofilms, particularly essential oils (EOs), which have long been recognized to have antimicrobial properties and are classified as “Generally Recognized as Safe” (GRAS) by the Food and Drug Administration (FDA). Due to their natural origin, their utilization in food formulations is supported by a positive public perception of them being a safer and more eco-friendly alternative to “synthetic agents” (Martillanes et al. 2017). EOs counteract the growth of bacteria, yeasts, and molds and have been extensively tested in vitro against a wide range of pathogenic bacteria and fungi, and in vivo for the control of potential pathogens in the animal gastrointestinal tract (Petretto et al. 2014). The activity of EOs is due to their phenolic compounds, such as coumarins, lignans, flavonoids, anthocyanins, tannins, quinines, and stilbenes. These compounds have been proven to be active against fungal biofilms and are less likely to induce resistant phenotypes. Thus, their use improves of the shelf-life of perishable products and the production of food without the use of synthetic additives. Tenore et al. (2012) showed that the polyphenolic fractions from red pitaya (*Hylocereus polyrhizus*) inhibited yeasts and molds. Shahzad et al. (2014) investigated the anti-biofilm activity of 14 polyphenols, of which pyrogallol (present in cocoa, coffee, and beer) and curcumin (available in common foods such as dried turmeric and curry power) displayed activity against *C. albicans* biofilms. Similarly, methanol olive leave extracts, rich in polyphenols, such as oleuropein, exerted strong antibiofilm activity against *C. albicans* (Takó et al. 2020). The anti-biofilm activity of terpenes against fungi and yeast have been reported by (Girardot and Imbert 2016). More recently, the anti-biofilm activity of aldehydic terpenes against the yeast *Cryptococcus neoformans* was reported by Kumari et al. (2019). Another recent study showed that essential oils from clove and thyme were able to efficiently inhibit biofilm formation by 17 *Candida* spp. isolated from different food matrices (Rajkowska et al. 2019). Tannins are other natural molecules that can be used to inhibit fungal biofilm; these substances also exhibit antioxidant, enzymatic inhibition, antidiarrheal, and mostly antiseptic properties, hence the interest in tannins as food additives (Bruneton 1999). Alejo-Armijo et al. (2017) showed that two procyanidins isolated from a laurel wood extract inhibited the growth of *C. albicans* at high concentrations and prevented biofilm formation at lower concentrations. Glasenapp et al. (2019) showed that the hydrolysable tannin fraction of Mangrove exhibits an anti-adhesion activity against *C. albicans*. Quinones also demonstrate antibacterial and fungicidal properties (Girardot and Imbert 2016). Tsang et al. (2012) showed that the quinone purpurin was able to repress yeast-to-hyphal transition in *C. albicans*, downregulating the expression of hypha-specific genes. In the context of natural products, hydrosols, complex mixtures containing traces of EOs and several water-soluble components, are also of significant interest (D’Amato et al. 2018). Plant extracts with unknown composition also showed antibiofilm activity against *C. albicans* biofilm. Such was the case for fresh garlic extract, *Cassia spectabilis* methanol leaf and crude extract, and methanol and ethyl acetate methanol extracts from *Schinus terebinthifolius* and *Croton urucurana* against *C. albicans* biofilm in in vitro studies (Shuford et al. 2005; Sangetha et al. 2009; Barbieri et al. 2014).

Besides plant extracts and EOs, the antimicrobial activity of small molecules against yeast biofilm formation has also been evaluated (Bou Zeidan et al. 2013). In particular, Bou Zeidan et al. (2014) reported the reduction of biofilm formation by *S. cerevisiae* when l-histidine was added as the sole nitrogen source. Similarly, Szafranski-Schneider et al. (2012) found that l-histidine modulates biofilm formation in *C. albicans*. Zara et al. (2019a) found that the addition of histidine, arginine, lysine, cysteine, tryptophan, phenylalanine, and threonine all reduce biofilm formation in *S. cerevisiae*. In particular, arginine and cysteine were the most effective against biofilm formation. In accordance, Abd et al. (2014) found that N-acetyl cysteine inhibits and removes *C. albicans* biofilms. Sanna et al. (2012) showed that methionine induces morphology changes in *Pichia fermentans* through the activation of a putative methionine-sensing machinery involving phospholipase C (Sanna et al. 2014).

Another interesting approach to the fight against fungal biofilm involves interfering with the normal functioning of quorumsensing molecules (QSMs). QSMs have been described in bacteria and in yeast, where they allow cell-cell communication among the members of a microbial community (Albuquerque and Casadevall 2012). In particular, *Candida*secreted QSMs induce phenotypic adaptations that include morphological changes, the secretion of virulence factors, and biofilm formation (Kruppa 2009; Deveau and Hogan 2011). Farnesol and tyrosol are the two most studied QSMs. Farnesol accumulation can block the yeasthyphal transition of *C. albicans* at high cell densities, thus preventing biofilm formation (Mosel et al. 2005). Recently, the effect of farnesol against *C. tropicalis* and other yeasts isolated from fruit juice filtration membranes in mono- and multispecies biofilms was assessed (Agustín et al. 2019). Similarly, the combination of tyrosol with other antifungals (amphotericin B, itraconazole, and fluconazole) showed a synergistic effect against *C. albicans* and *C. tropicalis* biofilms (Sebaa et al. 2019). By contrast, nitric oxide (NO), a recognized QSM, enhanced the biofilm formation of *S. cerevisiae* through *FLO11*-independent mechanisms (Yan and Bassler 2019). Finally, microbial derived molecules (killer toxins) have also been considered for the inhibition of yeast biofilm formation, but the efficacy of their effect remains rather controversial. While it has been suggested that the spatial use by biofilms represents a sort of protective armor against yeast killer toxins, other authors observed that *Aureobasidium*, *Pseudozyma*, *Ustilago*, and *Candida* spp. exert an extensive biofilm inhibitory effect on *Candida* that is very likely due to the secretion of killer toxins (Mannazzu et al. 2019).

## Conclusions

Recent scientific evidence has shown that yeast biofilms constitute a serious economic and health issue in food manufacturing. In addition, it is now well-ascertained that in many or most natural environments and foods, biofilms are formed by mixed bacterial, yeast, and fungal populations. For these reasons, one of the major challenges in current food microbiology is the identification of novel tools capable of preventing or removing mixed species biofilms. In this respect, the knowledge of microbial interactions and the identification of the genetic and environmental mechanisms involved in yeast biofilm formation could lead to the development of new biocontrol agents. Since the effectiveness of plant essential oils, quorum-sensing molecules, small molecules, and killer toxins has mostly been demonstrated in vitro using over-simplified systems, further studies are required to directly assess their activities on food matrices and the environment.
